# Hypoxia-Inducible Factor 1-α in Autoimmune Diseases—Insights from the Paradigm of Hashimoto’s Thyroiditis: A Narrative Review

**DOI:** 10.3390/medsci14010061

**Published:** 2026-01-28

**Authors:** Nika Srb, Andrea Milostić-Srb, Lea Sarić, Dubravka Holik, Matej Šapina, Rajko Fureš, Jasminka Talapko, Ivana Škrlec, Darko Katalinić, Borna Kovačić

**Affiliations:** 1 Medical Faculty Osijek, Josip Juraj Strossmayer University of Osijek, 31000 Osijek, Croatia; 2 Faculty of Dental Medicine and Health, Josip Juraj Strossmayer University of Osijek, 31000 Osijek, Croatiaiskrlec@fdmz.hr (I.Š.); 3 Health Center Osijek-Baranja County, 31000 Osijek, Croatia; sariclea97@gmail.com; 4 Clinical Hospital Centre Osijek, 31000 Osijek, Croatia

**Keywords:** autoimmune diseases, autoimmune thyroiditis, Hashimoto’s thyroiditis, HIF, hypothyroidism, hypoxia-inducible factor, narrative review

## Abstract

**Background/Objectives:** Given the rising prevalence of autoimmune diseases and the need for new insights into the pathology of these disorders, it is important to summarize current knowledge, with an emphasis on Hashimoto’s thyroiditis (HT), since it is especially on the rise. Hypoxia is part of various pathophysiological conditions, and hypoxia-inducible factor (HIF) is a key factor in these processes. Hypoxia is involved in the regulation of hormones and the development of endocrine disorders. With this in mind, this narrative review summarizes the current state of knowledge on the relationship between autoimmune diseases, focusing on HT and the effects of hypoxia through the role of HIF. **Methods:** Multiple databases such as PubMed, NIH, Scopus, Web of Science, ScienceDirect, and Google Scholar were thoroughly searched for relevant keyword. **Results:** In HT, thyrocyte-derived reactive oxygen species and chronic lymphocytic infiltration stabilize HIF-1α, tilting CD4+ T cell polarity towards Th17 and away from regulatory T cells. Increased levels of Mammalian target of rapamycin (mTOR)/HIF-1α and reduced Sirtuin 1 (SIRT1) in T cells from patients diagnosed with HT confirm this metabolic skew. Furthermore, the data position HIF-1α as a therapeutic target. Inhibitors of mTOR or agents that restore SIRT1 could complement levothyroxine and antioxidant strategies. Hypoxia and the HIF signaling pathway have a role in energy homeostasis through various ways, for example, via metabolic effects of thyroid hormones, which are associated with the clinical manifestations of HT. **Conclusions:** Elucidation of *HIF-1α*-centered gene networks and testing of HIF-targeted interventions may curb the growing clinical burden of HT.

## 1. Introduction

Autoimmune diseases are inflammatory disorders characterized by an elevated and dysregulated immune response against self-antigens. This leads to cell and tissue damage in various organs and can result in multiorgan failure [1,2]. Depending on where the target of attack is located, autoimmune diseases can be divided into systemic or organ-specific diseases [3]. In systemic autoimmune diseases, autoantigens can be found in almost all cell types [3]. Such diseases include systemic sclerosis (scleroderma), systemic lupus erythematosus (SLE), Sjögren’s syndrome, rheumatoid arthritis (RA), dermatomyositis, and anti-neutrophil cytoplasmic antibodies (ANCAs)-associated vasculitis [1,3]. In organ-specific autoimmune diseases, the patient’s immune system is primarily directed against a specific organ or tissue [3]. Such examples are targeting the skin in patients suffering from vitiligo or targeting the thyroid gland in those with Graves’ disease or Hashimoto’s thyroiditis (HT) [3,4]. The worldwide incidence of autoimmune diseases is 19.1% and the prevalence is 12.5%, confirming an overall increasing global trend [5]. HT is one of the most common thyroid diseases, with an incidence of 0.3–1.5 cases per 1000 people, and the incidence has been increasing rapidly over the past three decades, with the prevalence of the disease increasing with age, and is four times higher in women than in men [6,7,8]. As more and more diseases are discovered that have laboratory or clinical features associated with immune system involvement and autoimmune signatures, the number and scope of autoimmune diseases and chronic inflammatory disorders continue to increase, in addition to the currently recognized autoimmune diseases [5].

The etiopathogenesis of autoimmune diseases is attributed to a genetic predisposition, environmental influences, hormonal factors, and infections [1]. Epigenetic mechanisms, in which environmental influences can change how genes are expressed, even when the genetic code remains unchanged, are also important and provide information about the development of autoimmune diseases [1]. These epigenetic modifications act like molecular switches that turn genes on or off, and the transition may be flipped by different environmental factors such as diet, pollutants, and stress. In autoimmune diseases, epigenetic modifications can cause immune response dysregulation, leading to autoantibody production and damaging healthy tissues [1].

Recent findings have shed light on the role of hypoxia-inducible factor 1α (HIF-1α) in the development and pathology of various autoimmune diseases [9]. There is evidence (based on animal models and patients from clinical trials) that HIF-1α is responsible for regulating the secretion of inflammatory cytokines, leading to an imbalance of immune cells, such as Th1, Th2, Th17, Treg cells, and CD8+ T cells involved in autoimmune disorders [9]. These pro-inflammatory cytokines are released during adaptive immunity, and HIF-1α also affects cell differentiation and proliferation [9]. However, nearly all of these studies focus on systemic autoimmune diseases like RA and hardly any focus on the impact on Hashimoto’s thyroiditis [10,11]. Single-nucleotide polymorphisms of a gene also play a role since they may have a protective function against a disorder or they may be a risk factor for the disorder [12].

Therefore, this narrative review aims to evaluate the putative role of HIF-1α activation in driving thyroid-hormone imbalance and metabolic dysregulation in HT as well as the impact of polymorphism of the *HIF-1α* gene.

## 2. Methods

Studies that described the effect of *HIF-1α* on autoimmune disorders were included, with a specific focus on those that researched Hashimoto’s disease. Then, the impact of polymorphisms of the aforementioned gene on autoimmune diseases was examined, again with HT as the main search goal. Multiple databases such as PubMed, NIH, Scopus, Web of Science, ScienceDirect, and Google Scholar were thoroughly searched for relevant keywords “HIF-1α and autoimmune diseases”, “HIF-1α and Hashimoto’s thyroiditis”, “*HIF-1α* polymorphisms”, “*HIF-1α* polymorphisms and autoimmune diseases”, and “*HIF-1α* polymorphisms and Hashimoto’s thyroiditis”. Information on the polymorphisms was also obtained from the National Library of Medicine, HaploReg v4.2, Genotype-Tissue Expression (GTEx) Portal, Ensembl genome browser 115, and the GWAS Catalog. Since not many studies fit the inclusion criteria, the timeframe was not specified. The comprehensive search was for studies published in English, and reference lists of retrieved articles were screened to identify additional relevant studies. Both preclinical (in vitro studies: in cells or tissues and in vivo studies: animal models) and clinical trials were included in the review. Some of the included studies even combined both approaches. The studies were not excluded based on the number of participants. Studies were excluded if they focused primarily on *HIF1* polymorphisms and cancers, cardiovascular, infectious, or respiratory diseases or any other kinds of disorder, instead of autoimmune diseases.

## 3. Hypoxia-Inducible Factor (HIF)

### 3.1. HIF-1 in Hypoxic vs. Normoxic Environments

When cellular development and metabolism demand exceeds oxygen supply, cells and/or tissues often become hypoxic [13]. The pathophysiology of various human diseases, such as cancer, diabetes, aging, and stroke/ischemia, is significantly affected by hypoxia [13,14]. In physiological and pathological processes, hypoxia is mediated by the hypoxia-inducible factor (HIF) since oxygen is necessary for tissue functioning [15,16]. The three types of HIF are HIF-1, HIF-2, and HIF-3 [17,18,19]. HIF-1 is a heterodimeric transcription factor that consists of an oxygen-sensitive alpha subunit (HIF-1α) and an oxygen-stable beta subunit (HIF-1β) [15,17,20]. HIF-1α is expressed in an oxygen-dependent manner and is the active and regulatory part located in the q21–24 region of chromosome 14 [17,20]. HIFα has three different subunits (HIF-1α, HIF-2α, and HIF-3α), of which HIF-1α is ubiquitously expressed while the expression of HIF-2α and HIF-3α varies depending on the type of tissue cells [15,20]. The HIF-1β, dubbed the aryl hydrocarbon receptor nuclear translocator (ARNT), is constitutively expressed and is resistant to hypoxia. Therefore, the three beta subunits (HIF-1β, HIF-2β, and HIF-3β), are referred to as ARNT1, ARNT2, and ARNT3 [20]. HIF-1α and HIF-1β have an N-terminus that includes the PER-ARNT-SIM, abbreviated as PAS-A and PAS-B domains, and the basic helix–loop–helix (bHLH) domain [17,21,22]. These domains then form heterodimers with the α and β subunits, which are then recognized and bound to the DNA-binding site of HIF to assemble a nuclear dimer [17,21,22].

**Figure 1:**
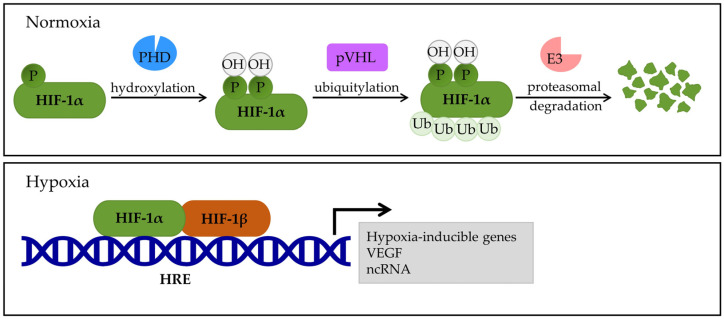
Effects of HIF-1α under normal oxygen conditions and oxygen deprivation. Under normal oxygen conditions, the prolyl hydroxylase domain protein (PHD) recognizes and hydroxylates proline residues in the oxygen-dependent degradation domain (ODDD) of the α-subunit of HIF. The von Hippel–Lindau protein (pVHL) recognizes the hydroxylated proline residues and ubiquitylates the HIF-1α subunit. The HIF-1α is then degraded by the E3 ubiquitin ligase in the ubiquitin–proteasome system. In hypoxia, the activity of PHD is reduced, and degradation of the HIF-1α subunit does not occur, but heterodimerization with the HIF-1β subunit occurs. The HIF-1α and HIF-1β heterodimer attaches to hypoxia response elements (HREs) and stimulates the transcription of numerous target genes, including VEGF (vascular endothelial growth factor) and ncRNA (non-coding RNA). PHD—prolyl hydroxylase domain protein, pVHL—von Hippel–Lindau protein, HRE—hypoxia response elements, VEGF—vascular endothelial growth factor, and ncRNA—non-coding RNA.

In normoxia, the oxygen-dependent degradation domain (ODDD) regulates HIF-1α stability via the hydroxylation of the proline residue [15,23]. Enzymes, such as Egl-9 Family Hypoxia-Inducible Factor (EGLN1, 2, and 3) or prolyl hydroxylase domain-containing protein (PHD)1, 2, and 3, hydroxylate the α subunits at conserved proline residues (p.Pro402 and p.Pro564), which then regulates the extent of HIF-1 activity [15,17]. Then, the von Hippel–Lindau protein complex (pVHL) recognizes the hydroxylated proline residues of HIF-1α and marks it for proteasomal degradation by E3 ubiquitin ligase [15]. The pVHL targets and ubiquitinates the N-terminal transactivation domain (N-TAD) within the ODDD domain, thereby controlling the proteasomal degradation of HIF-1α [15,17] (Figure 1). Even under normoxia, HIF-1α becomes stable when the ODDD domain is removed, resulting in HIF-1α heterodimerization, DNA binding, and transactivation that occurs independently without hypoxia signaling support [15,24]. The C-transcriptional activation domains (C-TADs) are transcriptional activation domains located at the C-terminus and are involved in fine-tuning transcription [17]. After forming a heterodimer with HIF-1β, HIF-1α binds to hypoxia response elements (HREs) to trigger the transcription of HIF-1 target genes [17,25].

### 3.2. HIF and Cell Signaling Pathways

HIF induces over 100 target genes in different cell types and is associated with 20 signaling pathways [15,20,26]. HIF-1 can only be stably expressed in hypoxic environments [20,27]. HIF-1α is involved in cell glucose metabolism by switching cells from the tricarboxylic acid cycle (TCA) to glycolysis under hypoxic conditions and has a role in the regulation of lipid metabolism and glucose catabolism [15,17]. Hypoxia causes vascular responses by stimulating angiogenesis and inducing the synthesis of vascular endothelial growth factor (VEGF), which is involved in angiogenesis and causes various diseases [15,17]. Aside from the control of protein-coding gene expression, HIF-1α has also been found to regulate noncoding RNA genes (ncRNA), such as transcribed-ultraconserved regions (T-UCRs) and microRNAs (miRNAs) [15].

Since HIF-1α is a transcription factor responsible for the expression of several genes, it is not surprising that it is regulated by multiple signaling pathways, including the PHDs/HIF-1α/pVHL; Janus Kinase/signal transducers and activators of transcription mitogen-activation protein kinase (JAK/STAT MAPKs); and phosphoinositide 3-kinase/protein kinase B/mammalian target of rapamycin pathway (PI3K/AKT/mTOR) [27]. Table 1 shows HIF and the currently known associated signaling pathways.

**Figure 2:**
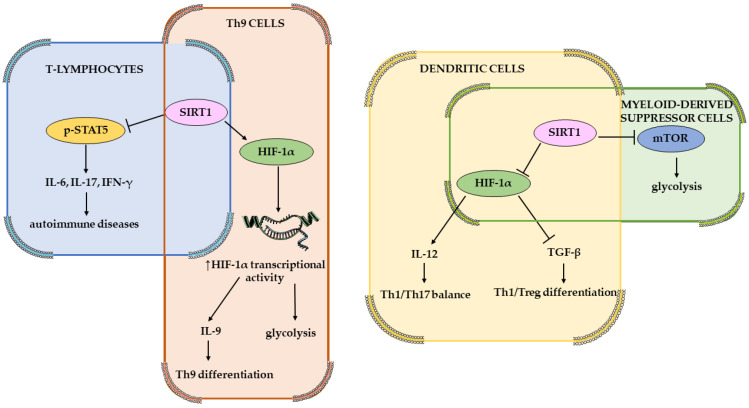
HIF–SIRT1–mTOR interplay within immune cell metabolism. T cell proliferation could be limited by SIRT1 by both downregulating signal transducer and activator of transcription 5 (STAT5) expression, thus suppressing pSTAT5 signaling. SIRT1 plays a crucial role in Th9 cell differentiation by regulating the glycolysis pathway and the mTOR-HIF-1α signaling pathway. Increased SIRT1 activity in response to phagocytic stimuli potentially modifies the Th1/Th17 balance during immunological disorders. When combined with the inhibitory effects of IFN-γ and IL-17 on pathogenic Th17 differentiation, specific deletion of SIRT1 in dendritic cells suppressed Th17 differentiation during inflammation. Adapted from Servier Medical Art “https://smart.servier.com (accessed on 10 December 2025)”, licensed under CC BY 4.0 “https://creativecommons.org/licenses/by/4.0/ (accessed on 10 December 2025)”. → mean activation and --| means inhibition.

In hypoxia, mitogen-activated protein kinase (MAPK), together with its subfamily, regulated extracellular protein kinases (ERKs) and activated HIF-1α, thereby promoting the development of cells and preventing oxidative stress-induced damage [25,27]. The epidermal growth factor (EGF) and insulin-like growth factor-1 (IGF-1) are also involved in activating the signaling pathway phosphoinositide 3-kinase (PI3K)/protein kinase B (PKB/AKT), which activates the target of rapamycin kinase (mTOR), thus promoting the expression of HIF-1α and leading to upregulation of VEGF expression, resulting in vasodilation [27,40,54]. The signal transducer and activator of transcription 3 (STAT3) activates AMP-activated protein kinase (AMPK), which inhibits the signaling pathway mTOR/HIF-1α, leading to a reduction in cell growth and a rise in programmed cell death [10,27]. Janus tyrosine kinase (JAK) can cause downstream phosphorylation and activation of STAT3, upregulating the expression of nucleotide-binding oligomerization structure-like receptor family pyrin domain protein 3 (NLRP3), thus exacerbating inflammation responses [61]. IL-6 interacts with the gp160 protein to activate the STAT3/HIF-1α signaling pathway, which regulates the differentiation of Foxp3^+^ regulatory T cells (Treg) [27]. Another interleukin is intertwined with the STAT3/HIF-1α pathway, namely IL-17, which is responsible for the regulation of autophagy and leads to inflammation and apoptosis of scar fibroblasts [10,27]. The upregulation of *HIF-1α* expression can also be activated by the binding of the tumor necrosis factor α (TNF-α) to the transforming growth factor-activated kinase 1 (TAK1), which stimulates glycolysis [27,62]. Hypoxic environments also induce high-mobility group protein 1 (HMGB1), which activates the c-Jun N-terminal kinase (JNK), thereby facilitating the HIF-1α/VEGF signaling pathway leading to angiogenesis [27,54]. The silent information regulator (SIRT), specifically SIRT1, attaches to the inhibitory domain of HIF-1α, thus protecting it from deacetylation; SIRT2, on the other hand, downregulates fibroblast phosphorylation [27]. Aside from this, SIRT1 also interacts with and deacetylates HIF-1α [63]. According to studies on mice, SIRT1 negatively affects mTOR, and HIF-1α is responsible for the glycolytic responses downstream of mTOR [64]. Also, SIRT1 and HIF-1α as metabolic sensors of oxygen have been shown to assist immune cells in adapting to the inflammation and changes in metabolism during immune responses [65,66]. Specifically, in the mouse model, mTOR and HIF-1α coordinated glycolytic metabolism during SIRT1-mediated myeloid-derived suppressor cell (MDSC) programming (Figure 2) [67]. In dendritic cells (again in mouse models), the interplays between SIRT1 and HIF-1α directed Th1 and iTreg cell differentiation under infectious inflammation by modulating the production of dendritic cell-derived T cell polarizing cytokines, such as IL-12 and TGF-β (Figure 2) [68]. Higher levels of thyroid autoantibody synthesis have been linked to abnormal SIRT1 expression in patients with autoimmune thyroid diseases [69]. Chronically activated CD4+ T cells, in HT patients, tend to express more mTOR and HIF-1α and less SIRT1 [37].

Prolyl hydroxylase domain-containing proteins (PHDs) belong to the superfamily of 2-oxoglutarate-dependent dioxygenases and regulate the levels of HIF [27,70]. Thus, when PHD is inhibited, HIF-1α is active and stable [70]. Prolyl hydroxylase inhibitors include thiamine deficiency, succinate, and oxidative stress, which stimulates the nuclear factor kappa-B (NF-κB), suppressing prolyl hydroxylases and, in turn, increasing HIF-1α [27].

## 4. HIF-1α and Autoimmune Diseases

The pathogenesis of autoimmune diseases may be caused by various processes, such as post-infectious immune dysregulation, chronic/latent recurring infection, or molecular mimicry [26]. The main feature of autoimmune diseases is tissue destruction activated by immune responses. Since the environment is hypoxic, it is no wonder HIF-1α may have a role in these diseases [26]. Table 2 shows autoimmune diseases in which the role of HIF has been demonstrated.

### 4.1. HIF-1α Effect on Immune Cells

HIF-1α is a pivotal metabolic sensor that binds to the hypoxia response element (HRE) found in the proximal region of the Rorc promoter, which serves as a coactivator for RORγt and contributes to the expression of IL-17a without directly binding to DNA on the IL-17a locus [97]. HIF-1α recruits the p300 factor to activate target genes, where p300 acetylates histones, opening the chromatin structure at the IL-17a gene promoter by binding RORγt, HIF-1α, and p300 [97]. A safe T cell inflammatory response is contingent upon initiating protective inflammation to eradicate the infection, terminating the inflammatory phase after removing invading components, and containing dysregulated or overaggressive immunological sequelae [26]. Autoimmune tissue damage may arise from T cells’ uncontrolled, self-aggressive inflammatory response [26,98]. Th17 is the most important T cell effector among the many T cell subsets in coordinating T cell-mediated autoimmune disorders [26,99]. In physiological settings, regulatory T cells (Treg) inhibit the expansion and autoimmune activity of effector T cells, particularly Th17 cells, which is not the case in autoimmune diseases since the balance is tipped [26,100]. In autoimmune diseases, IL-6 and TGF-β ensure the induction of naïve T cells in Th17 cells, and TGF-β and IL-2 ensure the induction of naïve T cells in Treg cells [26,101]. The TGF-β also increases the expression levels of transcription factors, including RORγt and FOXP3 [26]. Thus, the suppression of FOXP3 simultaneously with a continued expression of RORγt is key for the differentiation of pathogenic Th17 cells [26].

When transcription of the *RORγt* gene is activated, it recruits p300, enhances the expression of Th17-related genes, and drives Th17 cell differentiation while suppressing Treg differentiation by downregulating FOXP3 protein [102]. All this regulates the increase in the number of Th17 cells, the production of IL-17, and the increased activation and recruitment of T lymphocytes and macrophages [102]. HIF-1α is, therefore, a transcription factor for Th17. Since Th17 cells play a crucial role in many autoimmune disorders, changes in the expression of Th17 transcriptional regulators could be linked to the response of Th17 cells to disorders like psoriasis, rheumatoid arthritis, multiple sclerosis, Crohn’s disease, and inflammatory demyelinating disease [97,102]. Various transcriptional factors, including HIF, regulate the balance of Th17/Treg. An imbalance, therefore, causes the onset and advancement of many autoimmune diseases [102]. Treg cells in rheumatoid arthritis patients demonstrate decreased expression of STAT3 and HIF-1α [102]. The functions of HIF-1 in rheumatoid arthritis include promoting inflammation, angiogenesis, and cartilage destruction [17]. Research in animal models shows that hypoxia and HIF-1α enhance FOXP3 transcription, which favors Th17 over Treg cell differentiation [102]. Studies show that HIF-1 also has effects on Th1 cells. However, in mice, it appears to favor the development and activity of pathogenic Th17 cells [20,26]. In a hypoxic environment, HIF-1α suppresses the expression of STAT4, a critical transcription factor essential for Th1 cell development and function [20,26]. There is still a lot to discover about the exact role that HIF-1 plays in B cells in autoimmune disorders. However, it appears to be a suppressive role [20,26]. IL-10 initiates the production of regulatory CD1dhi CD5+ B cells, which have anti-inflammatory effects in autoimmunity [20,26]. When HIF-1 levels increase, it induces glycolysis, which leads to the triggering and amplification of CD1dhi CD5+ B cells [26]. If HIF-1 is missing, the regulatory B cells lose their ability to produce IL-10 effectively [26].

Thyroid disorders, especially Hashimoto’s thyroiditis, are more common in women compared to men. This effect is probably due to the immunostimulant properties of estradiol [103,104]. The ovary is a common target for autoimmune attacks, and polycystic ovarian syndrome is associated with various autoimmune disorders such as psoriasis, autoimmune thyroid disease, and Hashimoto’s thyroiditis [105,106]. Similarly, a study conducted in 2024 found that autoimmune polyendocrinopathy–candidiasis–ectodermal dystrophy (APECED) was connected to increased HIF-1α and reduced FOXP3 in polycystic ovarian syndrome patients with autoimmune thyroiditis [105]. Since autoimmune disorders tend to co-occur in the same patient, sometimes HT occurs with other autoimmune disorders, such as autoimmune polyendocrine syndromes [107,108]. Autoimmune polyendocrine syndromes (APSs) are a heterogeneous group of diseases in which immune-mediated destruction affects two or more endocrine glands. Of the three main types of APSs, HIF-1α is most strongly associated with type 3, which includes autoimmune thyroid diseases such as HT and autoimmune diseases that do not involve the adrenal glands (e.g., celiac disease, vitiligo, and pernicious anemia). Thyroid inflammation in APS3, as mentioned above, in the murine model is associated with activating the mTOR/HIF-1α signaling pathway in CD4+ T cells, which promotes the production of proinflammatory cytokines and metabolic dysregulation [109,110].

### 4.2. Single-Nucleotide Polymorphisms and Autoimmune Diseases

SNPs or single-nucleotide polymorphisms are genomic variants of single-base (adenine, thymine, cytosine, or guanine) positions in the DNA [111,112]. The prevalence of 1% makes them the most common type of genetic variation that also increases the risk of developing a disease or disorder and, as such, are useful biomarkers [111,112]. *HIF-1α* gene polymorphisms often lead to chronic obstructive pulmonary disease, cardiovascular disease, skin diseases, and complications of diabetes mellitus [113]. Such diabetic complications include nephrotic disorders [114]. *HIF-1α* polymorphisms predispose systemic sclerosis, confirming HIFs’ role in the disease pathology and as a biomarker [115,116]. A study from 2017 linked a higher receptiveness to autoimmune thyroid diseases in patients with SNPs like rs12976445 and rs3746444 [117]. Feng et al. were unable to establish that the four *HIF-1α* polymorphisms studied from patients (rs11549465, rs12434438, rs1957757, and rs1951795) were associated with systemic lupus erythematosus [118]. However, a study in 2021, also on human patients, found that the *HIF-1α* (rs11549465) CT polymorphism is a protective factor for systemic lupus erythematosus susceptibility [40]. Another research reports that the same SNP (rs11549465) was overexpressed in psoriasis patients [119]. A protective effect of that polymorphism was established against diabetes mellitus types 1 and 2 in a Caucasian sample [120]. The rs11549465 polymorphism, namely the substitution of cytosine at position 1772 with thymine, results in a missense mutation, namely the substitution of proline at position 582 with serine at the protein level (Pro582Ser or P582S) [121]. This polymorphism is located within the oxygen-dependent degradation domain (ODD) of the HIF-1α protein, which is important for its stability and regulation. Although this change does not affect the hydroxylation of the proline residue at position 564 of HIF-1α, it creates a molecule that resists degradation, making it more prone to nuclear translocation, and therefore the products of this transcriptional activity have a protective effect. Similarly, the rs11549467 polymorphism results in a missense mutation that causes a substitution of alanine at position 588 with thymine (A588T) at the protein level. It is located in exon 12 of the *HIF-1α* gene and within the ODD domain [122]. The SNP (rs11549467) was associated with systemic sclerosis in patients with the disease [115].

## 5. Hashimoto’s Thyroiditis

The most common organ-specific endocrine disorders are autoimmune thyroid diseases, with an estimated prevalence of 3% to 5% [6,123]. Hashimoto’s thyroiditis (chronic lymphocytic or autoimmune thyroiditis) is an autoimmune disease characterized by an enlarged thyroid gland, heavy infiltration of lymphocytes in the parenchyma, specific autoantibodies in the serum such as thyroid peroxidase antibodies (TPOAbs) and thyroglobulin antibodies (TgAbs), and the most common form is the T cell-mediated organ-specific chronic inflammation type [6,123,124]. In general, women (5% to 15%) are more commonly affected by autoimmune thyroid diseases than men (1% to 5%), and the prevalence of HT varies significantly based on geographic location [8,125]. With the progression of the disease, the thyroid gland is continuously destroyed, causing abnormal gland function, which leads to hypothyroidism in about 20% to 30% of patients [124]. Thus, HT is the most common cause of hypothyroidism worldwide [6]. The development of HT is caused by multiple factors, such as genetic susceptibility, epigenetics, and/or environmental factors [126].

The standard diagnosis includes evaluating the clinical features of HT, serologic detection of autoantibodies against thyroid antigens (thyroid peroxidase and thyroglobulin), and an ultrasound scan of the thyroid gland [127]. Due to their sensitivity and specificity, anti-thyroperoxidase antibodies (TPOabs) are the most effective serologic marker for HT diagnosis since they are detectable in around 95% of HT patients and rare in healthy controls [127]. Anti-thyroglobulin antibodies (Tgabs) are antibodies against the most abundant protein in the thyroid gland and are present in a more significant number and proportion in healthy controls. Therefore, Tgabs are less sensitive and present in 60% to 70% of HT patients [127]. Some patients may exhibit extremely high antibody titers, significant changes in thyroid texture, and constant thyroid function abnormalities, all leading to fluctuations in thyrotropine (TSH) levels and causing alternation between hypothyroidism and hyperthyroidism [124].

### 5.1. Pathophysiology of HT

The pathology of HT includes antibody-producing B lymphocytes and CD4+ T lymphocytes that stimulate Th1 cytokines. Thus, the main histologic characteristic of HT is the destruction of follicles and infiltration of lymphatic and plasma cells into the thyroid gland [128]. These Th1 cytokines (e.g., IL-1α, IL-2, IL-1β, IFN-γ, TNF-α, and TNF-β) then lead to the destruction of thyrocytes by CD8+ cytotoxic cells, causing epithelial cell destruction fibrosis and gland atrophy and consequently primary hypothyroidism [62]. Studies on transmission electron microscopy found that the thyroid follicles tend to be smaller than average, have limited colloid, as well as endothelial destruction in the larger blood vessels [128]. Another key factor for cell destruction is oxidative stress, caused by increased H_2_O_2_ and superoxide anion production by NOX4, which is found in thyrocytes [129]. The thyrocytes adapt by growing levels of HIF-1α, which upregulates glucose transporter 1 (GLUT-1), leading to NOX4-related reactive oxygen species production [130]. The study conducted by Hepp et al. found that SIRT1 downregulation correlates with HIF-1α upregulation in Th1 cytokines of patients with HT [45]. They also concluded that a similar correlation can be deduced between the upregulation of VEGF-A and HIF-1α in Th1 cytokines from patients with HT, and therefore proposed a pathogenic pathway for developing HT based on the production of reactive oxygen species and downregulation of SIRT1, and pointed out the therapeutic possibilities of SIRT1 as an antioxidant [45].

The thyroid hormones play a crucial role in regulating energy metabolism. The most important hormone secreted by the thyroid gland is thyroxine (T4). In the target tissues, T4 can be converted into the active form triiodothyronine (T3) or into the inactive form, the reverse T3 (rT3) [131]. Iodothyronine deiodinases mediate the conversion of T4 into T3 or rT3. In different states, thyroid hormones are regulated by activating deiodinase (D2) and inactivating deiodinase (D3), and the regulation of D2 and D3 in target tissues determines thyroid hormone function. Hypoxia induces D3 expression in a HIF-dependent manner [132]. Elevated D3 levels reduce T3 levels and disrupt mitochondrial energy homeostasis, which can lead to cell death [133]. Although there is little research linking hypoxia, HIF, and HT, hypoxia influences the action of thyroid hormones and is important for the energy homeostasis of the organism.

### 5.2. Hashimoto’s Encephalopathy

Hashimoto’s encephalopathy (HE), also called steroid-responsive encephalopathy associated with autoimmune thyroiditis (SREAT), is an autoimmune disorder with heterogeneous presentation and unclear pathogenesis [134]. HE is characterized by behavioral abnormalities, cognitive abnormalities, dementia, tremor, generalized and partial seizures, status epilepticus, headaches, hallucinations, delusions, paranoia, and psychosis [135,136,137]. Since HE is an autoimmune disorder, there is no wonder it has been associated with increased levels of anti-thyroperoxidase (anti-TPO), anti-thyroglobulin antibodies (anti-Tgs), thyroid peroxidase antibodies (TPOAbs), anti-thyroglobulin antibodies (Ab anti-Tgs), anti-nuclear antibodies (ANAs), rheumatoid factor (RF), and C-reactive protein (CRP) [138,139,140,141], while thyroid hormone levels may vary, hence the heterogenous presentation (euthyroid, hypothyroid, or hyperthyroid) [134]. A study by Tani et.al on autopsy cases suggest that elevated thyroid-related hormones (T3 and T4) levels in the pathophysiological field could point to systemic hypoxia/ischemia [142]. Elevated levels of cerebrospinal fluid proteins and anti-amino (NH2)-terminal of α-enolase antibodies, which have been linked to the nervous system, also occur [134]. Numerous studies have observed the role of HIF-1 in the pathology of CNS-related diseases (e.g., intracranial atherosclerosis, Alzheimer’s disease, and subarachnoid hemorrhage) [143,144,145,146]. Since various antibodies and proteins are elevated in SREAT, specifically those in the nervous system, and HIF-1 has been associated with neurological pathophysiology, it may be a potential biomarker as well as a therapeutic target [147]. Treatment of HE consists mainly of corticosteroids, although other types of treatment include intravenous immunoglobulins, immunosuppressants (e.g., methotrexate), rituximab, and levothyroxine [134,148,149,150,151]. Studies on cell lines suggested that activation of *HIF-1α* induced by hypoxia leads to amplified inflammation and corticosteroid resistance [152]. A 2025 study on rat models found that glucocorticoids may inhibit osteoblasts’ osteogenic differentiation by activating the HIF-1α/VEGF signaling pathway [153]. A study on human and mouse tissue samples suggested that by impairment of HIF-1α-dependent glucose absorption in activated macrophages, glucocorticoids reduce inflammation [154].

### 5.3. Role of HIF-1α in Hashimoto Thyroiditis

Zhao et al. performed a study in 2021 to examine mTOR/HIF-1α and SIRT1 levels in the CD4+ T cells of rodents with subacute thyroiditis, aiming to understand abnormal metabolic pathways and identify key contributors to metabolic dysregulation [37]. The study observed chronically activated CD4+ T cells in HT and found a decreased SIRT1 expression and increased levels of HIF-1α and mTOR, leading them to conclude that the inactivation of SIRT1 might play a role in modulating the metabolic processes in CD4+ T cells [37]. Mice exhibited increased levels of mTOR and HIF-1α, which were reduced following treatment with 2-Deoxy-D-glucose (2DG) and/or metformin, and the expression of SIRT1 went back to normal [37]. The usual treatment of HT comes down to treating hypothyroidism; thus, drugs like levothyroxine are the most common type of treatment [155,156]. However, treatment also depends on the clinical presentation of HT. HT with euthyroidism, as well as HT with subclinical hypothyroidism, does not require treatment [157]. Overt hypothyroidism is treated with levothyroxine, as already mentioned [155,156,157]. Nonsteroidal anti-inflammatory drugs can be used (although rarely) as pain relief for thyroid pain and tenderness [158]. Since selenium reduces levels of thyroid antibodies and TSH due to its antioxidative and anti-inflammatory role, HT patients may benefit from using selenium supplementation [159,160].

Since genetic instability (e.g., SNPs) is a highly adverse prognostic factor for thyroid carcinomas, genomic sequencing techniques are useful for identifying them [161,162]. The risk of developing thyroid cancer increases in individuals with autoimmune thyroid diseases and correlates with the degree of inflammation and obesity [163]. Individuals with an elevated body mass index might have an increased risk of developing HT. Obesity, for example, might contribute to HT development and influence the course of the autoimmune disease, but hypothyroidism may also increase body weight. Thyroid function could be one of the factors influencing body weight and the comorbidities of obesity [164]. For example, HIF-1α activity has been shown to rise in adipose tissue, contributing to chronic inflammation in obesity. In mouse models, several signals, such as hypoxia, insulin, and adipogenesis in obesity, influence HIF-1α activity in adipose tissue [165]. For example, the T allele of the rs2057482 polymorphism is linked to a reduced risk of obesity in individuals at higher risk of premature coronary artery disease [166]. Moreover, thyroid hormones are important in the bidirectional relationship between obesity and thyroid immunity. Changes in thyroid hormone levels can promote obesity, atherosclerosis, and inflammation. For example, low T4 levels in hypothyroid patients can contribute to inflammation and activation of the immune responses [167]. The effect of T4 occurs via an extragenomic pathway that can modulate the immune responses by activating various, different signaling pathways, including HIF-1α [168]. The effects of thyroid hormones on the immune responses are indirect and may be mediated by a combination of genomic and non-genomic actions in particular cellular/clinical contexts. This is particularly important in obese patients, who often have reduced T4 levels [163].

Obstructive sleep apnea (OSA) is a persistent condition distinguished by periodic breathing interruptions during sleep, resulting in intermittent oxygen deprivation. *HIF-1α* is crucial for maintaining homeostasis of oxygen metabolism [169]. Research on patients has established a link between the development of OSA and HT, with evidence of a bidirectional relationship in which OSA may increase the risk of autoimmune diseases, including HT [170]. Notably, serum levels of HIF-1α are more increased in individuals with OSA than in controls, suggesting that HIF-1α may contribute to various comorbidities associated with OSA [169]. In addition, hypoxia-induced cell damage in OSA could trigger an enhanced immune response due to increased exposure to antigen-presenting cells. Furthermore, individuals with elevated serum TSH levels had higher Epworth Sleepiness Scale scores than those with normal TSH levels [171]. Moreover, some studies have shown that specific polymorphisms of the *HIF-1α* gene are associated with OSA. In individuals with the TT genotype of the *HIF-1α* gene’s rs11549465 (C1772T) polymorphism is linked to severe OSA [172]. It was also found that carriers of the T allele and the CT genotype of the same polymorphism have an increased risk of obstructive sleep apnea–hypopnea syndrome in the Russian population [173].

### 5.4. HIF-1α as a Therapeutic Target

A study conducted in 2024 researched the link between thyroid-associated ophthalmopathy, which is associated with Graves’ disease, and autoimmune hypothyroid patients, and HIF-1α because of its role in adipogenesis and fibrosis [174]. The researchers concluded that significantly higher HIF-1α expression was found in these patients, thus suggesting HIF-1α as a therapeutic target [174]. Research that studied the influence of HIF-1α and HIF-2α in patients with HT found that they play a role in the development of papillary thyroid carcinoma and highlighted this as a potential biomarker and thus a possible target for future drugs [174,175]. Metformin has been suggested as treatment for hypothyroid patients since it reduces TSH levels [176]. It has also shown to reduces the overactivation of the mTOR/HIF-1α/HK2/glycolysis pathway in HT examinees [37]. However, the known adverse effects (nausea, emesis, diarrhea, hypoglycemia, headaches, and diaphoresis) of the drug may also appear and it is contraindicated in patients with severe renal dysfunction [177]. In human case–control studies, vitamin D deficiency leads B-lymphocytes to proliferate and differentiate into plasma cells, which then secrete high levels of IgG and IgE, causing thyroid cells to be damaged and leading to HT [178]. Therefore, vitamin D supplementation has been suggested as alternative treatment therapy for HT. In HT examinees, vitamin D has been shown to have a positive correlation to TNF-α, IL-5, and IL-17 as well as the ability to inhibit IFN-γ, which is associated with the suppression of Tregs expression and the activation of CD8+ T cells [179,180]. In vitro studies on samples from patients with HT found that vitamin D has exhibited an anti-proliferative effect in Th cells that was blocked by SIRT1 inhibition, followed by an elevation of *FOXO3a* gene expression; therefore, the SIRT1-FOXO3a axis is one of the downstream targets of vitamin D immunoregulatory effects [181]. Meta-analyses have shown that studies about treating HT with vitamin D reported no adverse effects [182,183]. Although one study did report nausea, vomiting, and dermatitis, none of the toxicity complications such as hypercalcemia, hypercalciuria, or renal failure were reported [184,185].

## 6. Limitation of Current Evidence and Future Research Directions

Since some of the included studies and their findings mentioned in this review were performed on animals and some were performed on humans, the results cannot be compared. Also, the studies that were conducted on human patients all had different numbers of participants. In order to gain better insight, bigger studies, conducted on large numbers of patients, are needed. On the other hand, this is also one of the reasons for writing this review in order to highlight the need for more research on autoimmune disorders and the effect of polymorphisms on human examinees. However, some of the studies had their strength and took even a step further and conducted their research on both human tissue samples and animal models.

The growing body of evidence suggests that there is a link between *HIF-1α* and the incidence of autoimmune diseases. We suggest that future research focus on the role of *HIF-1α* polymorphisms in the occurrence of Hashimoto’s thyroiditis. As was mentioned in the paper, some SNPs have a protective effect against the occurrence of autoimmune diseases, while others lead to higher incidences of these diseases. Based on this, future studies should center on various polymorphisms in order to obtain better insight. Using technologies such as real-time polymerase chain reaction in order to obtain better insight into the effect and role of *HIF* polymorphisms in Hashimoto’s thyroiditis should be applied in future studies. Proteomics and single-cell sequencing can offer a more thorough comprehension of the mechanisms underlying hypoxia in many tissues and cells. Methods such as immunofluorescence microscopy to explore different patterns of subcellular localization of HIF-1α should be used, as well as mass spectrometry (MS) for studying HIF-1α by identifying post-translational modifications. Integrative genomics should also be applied for a holistic view of the genomic structure and function in order to identify novel HIF-1 target genes. The combination of single-cell DNA and RNA sequencing has been showing promising results in other fields and thus may be used in the study of HIF1 as well. Similarly, multiple cancers have been identified as HIF1 target genes by using ChIP-seq and RNA-seq datasets.

Here is what is currently known about Hashimoto’s thyroiditis and *HIF-1*. Hypoxia plays a crucial role in the pathophysiology of various human diseases, particularly through the activity of hypoxia-inducible factor 1 (HIF-1), a key transcriptional mediator of the hypoxic response. In both physiological and pathological processes, hypoxia is mediated by HIF, which activates over 100 target genes across different cell types and is involved in 20 signaling pathways. Notably, HIF-1 can only be stably expressed within hypoxic environments. In autoimmune diseases, the environment is hypoxic. Furthermore, single nucleotide polymorphisms (SNPs), the most common type of genetic variation, not only heighten the risk of developing certain diseases or disorders and as such serve as valuable biomarkers, but in the context of Hashimoto’s thyroiditis (HT), the pathology is marked by the presence of antibody-producing B lymphocytes and CD4+ T lymphocytes.

Here is a summary of what is yet to be explored and/or firmly confirmed. *HIF-1α* polymorphisms predispose autoimmune diseases. Increased levels of HIF-1α and mTOR can be found in chronically activated CD4+ T cells in HT. Decreased thyroxin levels in hypothyroid patients can contribute to inflammation and activation of the immune responses, which occurs via an extragenomic pathway that can modulate the immune responses by activating various signaling pathways, including HIF-1α. Increased HIF-1α expression was found in Graves’ disease patients, suggesting HIF-1α as a therapeutic target. Therefore, HIF-1α should also be considered as a therapeutic target in HT. Research that studied the influence of HIF-1α in patients with HT found that it plays a role in the development of papillary thyroid carcinoma and highlighted this as a potential biomarker and thus possible target for future drugs. Metformin has been suggested as treatment for hypothyroid patients since it reduces TSH levels and has also shown to reduce the overactivation of the mTOR/HIF-1α/HK2/glycolysis pathway in HT examinees.

## 7. Conclusions

Hypoxia occurs in almost all pathological disorders, and HIF plays an important role. Hypoxia-induced stabilization of HIF-1α is at the interface of metabolic stress, epigenetic reprogramming, and immunological dysregulation, typical of autoimmune diseases. By orchestrating Th17/Treg imbalance, modulating deiodinase activity, and promoting oxidative damage, HIF-1α represents a unifying mechanistic thread linking disparate clinical observations in HT with broader trends in systemic and organ-specific autoimmune diseases. The dual role of HIF-1α as a metabolic sensor and immune regulator underscores its potential as a therapeutic target. Recognizing this nexus not only clarifies the etiology of the disease but also opens tangible translational pathways as follows: pharmacological attenuation of HIF-1α (e.g., mTOR inhibitors, PHD agonists), restoration of counter-regulatory nodes such as SIRT1, and genotype-guided risk stratification promise more precise prevention and therapy. As various studies also emphasize the influence of *HIF-1α* single-nucleotide variations, research into their specific effects on HT is also needed. By linking molecular mechanisms to clinical findings, this review underscores the need to translate HIF-1α biology into personalized strategies for treating autoimmune thyroid diseases and their systemic sequelae.

## Figures and Tables

**Table 1:** HIF and associated pathways.

Pathway	Function and HIF-1α Interplay	References
Phosphatidylinositol 3-kinase/protein kinase B (PI3K/Akt)	Regulates HIF-1α levels by activating its transcription and translation (human cell lines); Affects the immune function of neutrophils (human patients).	[28,29]
Nuclear factor kappa-light-chain-enhancer of activated B cells (NF-κB)	HIF-1α promotes the expression of NF-κb-regulated inflammatory cytokines in macrophages after lipopolysaccharide stimulation (mouse models); HIF-1α mediates NF-κb activation in anoxic neutrophils (mouse models); NF-κb signaling is oxygen-dependent (human cell lines).	[30,31]
Signal transducer and activator of transcription 3 (STAT3)	STAT3 activates HIF-1 target genes by binding to specific HIF-1 target gene promoters (human cell lines and animal models—husbandry and zebrafish lines).	[32,33]
Neurogenic locus notch homolog protein 1 (Notch-1)	Notch and HIF-1α bind directly to each other in response to ischemia-like conditions (mouse models).	[34]
C-X-C motif chemokine ligand 12 (CXCL12)	HIF-1 and the CXCL12/CXCR4 axis regulate the interaction of chronic lymphocytic leukemia cells and the tumor microenvironment (human patients); In hypoxia HIF-1α regulates Cxcl12 in hepatocytes (mouse models).	[35,36]
Phosphoinositide 3-kinase/protein kinase B/mammalian target of rapamycin pathway (PI3K/Akt/mTOR)	PI3K/Akt/mtor enhances HIF-1α translation (human patients); Thyroiditis elevates the mtor/HIF-1α/HK2/glycolysis pathway in CD4+ T cells (human patients).	[37]
Vascular endothelial growth factor (VEGF)	VEGF is upregulated by HIF-1 (human patients).	[38,39,40]
Angiopoietin-like 4 (ANGPTL4)	HIF-1α regulates the expression of ANGPTL4 (rat models and human cell lines).	[41,42]
Receptor activator of nuclear factor kappa-Β ligand (RANKL)	HIF-1α promotes the expression of RANKL by activating JAK2/STAT3 pathway (mouse cell lines).	[43,44]
Janus Kinase/signal transducers and activators of transcription (JAK2/STAT3s)	HIF-1α promotes the expression of RANKL by activating JAK2/STAT3 pathway (mouse cell lines); HIF-1α expression activates the JAK2/STAT3 pathway (mouse cell lines).	[43]
Sirtuin 1 (SIRT1)	SIRT1 induces the deacetylation of HIF-1α, therefore inhibiting cellular accumulation of HIF-1α (human patients and mouse models).	[45,46]
Sirtuin 6 (SIRT6)	SIRT6 over-expression inhibits the transcriptional activation of HIF-1α/PDK4 signaling (rat models); SIRT6 increases glycolysis through the HIF-1α/HK2 (hexokinase-2) signaling pathway (human tissue).	[47,48]
Interleukin-17 (IL-17)	IL-17 induces expression of HIF-1α (mouse models and human tissue samples).	[49,50]
Interleukin-38 (IL-38)	IL-38 down-regulates HIF-1α (mouse models).	[51]
Extracellular signal-regulated kinase (ERK)	ERK1/2 regulates the nucleocytoplasmic transport of HIF-1α (human cell lines); ERK signaling controls productive HIF-1 binding to chromatin (human cell lines),	[52,53]
The target of rapamycin complex 1 (TORC1)	Mtorc1 mediates VEGF-A expression via HIF-1α (mice and human cell lines).	[54,55]
Transforming growth factor-beta (TGF-β)	HIF-1α is a critical factor in the dual role (inhibits glycolysis under normoxia while significantly promoting tumor cells’ glycolysis under hypoxia) of TGF-β in tumor cells (human cell lines).	[56,57]
Glucose transporter 1 (GLUT1)	HIF-1α induces GLUT1 expression (human patients, human tissue, and human cell lines).	[45,58,59]
Retinoic acid-related orphan receptor gamma t (RORγt)	HIF-1α induces expression of RORγt (mouse models).	[60]

**Table 2:** HIF’s role in different autoimmune diseases.

Autoimmune Disease	Role of HIF	Reference
Ankylosing spondylitis (AS)	Disruption of long non-coding RNA (LncRNA)-mediated competitive HIF pathways may be involved in the pathogenesis of AS (human tissue samples); Hypoxia in AS lesions could serve as a trigger for immune cells (neutrophils), to become activated and contribute to the pathogenesis of AS through the MAPK and HIF-1 signaling pathways (human tissue samples).	[71,72]
Atopic dermatitis (AD)	Using a decoy strategy, the inhibition of HIF-1α and STAT5 transcription factors effectively attenuates mast cell survival and alleviates AD-like skin disease in vitro and in vivo models (mouse models); Lack of HIF-1α and HIF-2α in the epidermis leads to dry skin, impaired permeability barrier, and elevated sensitivity to cutaneous allergens (mouse models).	[73,74]
Crohn’s disease (CD)	Inhibition of HIF-1α/ABC transporters restores Th17 response to unconjugated bilirubin in CD (human cell lines and mouse models).	[75]
Dermatomyositis (DM)	Vascular neogenesis in dermatomyositis patients is regulated by the VEGF/HIF pathway (human tissue samples); HIF-1α within the muscle fibers of dermatomyositis patients; hypoxia may contribute to the pathogenesis of DM (human tissue samples).	[76,77]
Graves’ ophthalmopathy (GO)	HIF-1-dependent pathways stimulate angiogenesis and adipogenesis, thus impacting tissue remodeling in GO (human tissue samples).	[78,79]
Hashimoto’s thyroiditis (HT)	Upregulation of HIF-1α in Th1 cytokines induces an increase in ROS and a decrease in antioxidants; Excessive iodine adsorption activates HIF-1α pathway to promote apoptosis of thyroid follicular cell (risk factor for HT development) (human tissue samples).	[45,80]
Inflammatory bowel disease (IBD)	Stabilization of HIF-1α through prolyl hydroxylase inhibition (PHDi) results in earlier and increased epithelial integrin β1 expression, leading to accelerated mucosal healing and restitution of epithelial barrier function (human cell lines in mouse model).	[81,82]
Myasthenia gravis (MG)	HIF-1α alleviates the inflammatory responses and rebuilds the CD4+ T cell subsets balance (rat models); Th17/Treg imbalance in myasthenia gravis may relate to increased HIF-1A (human tissue samples).	[83,84]
Multiple sclerosis (MS)	Elevated HIF-1α at mRNA and protein levels in the early lesions of MS (human tissue samples); Hypoxia leads to neuroinflammation and tissue expression of HIF-1α correlates with neurological deficits (rat models).	[85,86]
Psoriatic arthritis (PsA)	Serum levels of VEGF and HIF-1 were elevated in PsA patient groups (human patients); Stiffness-dependent lysyl oxidase regulation through HIF 1 leads to extracellular matrix modifications in PsA (human tissue samples).	[38,87]
Rheumatoid arthritis (RA)	JAK1/STAT3/HIF-1α pathway is essential for the proliferation of fibroblast-like synoviocytes and pannus formation (rat models); IL-33 induces HIF-1α expression in fibroblasts, forming a HIF-1α/IL-33 regulatory unit that perpetuates the inflammation process in RA (human tissue samples).	[10,88]
Sjögren’s syndrome (SS)	HIF-1α activation prevents dry eye (mouse models); HIF-1α can directly transcribe and activate RORγt to enhance Th17 development causing imbalance of T cell subsets and enhanced inflammation (human tissue samples); HIF-1α Pro582Ser T allele and C/T genotype polymorphism was identified as a genetic factor associated with SS (human tissue sample).	[89,90,91]
Systemic lupus erythematosus (SLE)	HIF-1α (rs11549465)-CT was identified as a protective factor (human tissue sample); HIF-1α contributes to the activation of Th17 cells in SLE (human tissue and mouse models); T cell-rich inflammatory infiltrate exhibited higher levels of HIF-1 and a cytotoxic signature (mouse models).	[40,92,93]
Diabetes mellitus (DM)	HIF-1α plays a key role in β cell reserve and regulation of ARNT expression (human tissue and mouse models); Hyperglycemia impairs hypoxia-dependent protection of HIF-1alpha against proteasomal degradation (mouse model); HIF-1 promotes wound healing by elevating angiogenesis and fibroblast proliferation; Protein stabilization and transactivation activity of HIF-1 are inhibited in wounds of patients with diabetes and in animal models.	[94,95,96]

## Data Availability

No new data were created or analyzed in this study.
